# Integration of bulk RNA-seq data with single-cell RNA-seq data to identify urinary biomarkers derived from prostate tumor tissues

**DOI:** 10.1186/s12894-026-02295-y

**Published:** 2026-08-03

**Authors:** Yanting Shen, Yuhan Guo, You Wu, Liyang Dai, Jican Liu, Ting Zhu, Junyan Xu, Huifeng Li, Rui Yang, Zhikang Cai, Xiawei Fei

**Affiliations:** 1 School of Gongli Hospital Medical Technology, University of Shanghai for Science and Technology, Shanghai, 200093 PR China; 2 School of clinical medicine, Shanghai University of Medicine & Health Sciences, Shanghai, 200237, PR China; 3 Department of Urology and andrology, Pudong Gongli Hospital, Shanghai University of Medicine & Health Sciences, Shanghai, 200135 PR China; 4 School of Medicine, Southeast University, Nanjing, 210009, PR China; 5 Department of Urology, Qingpu Branch of Zhongshan Hospital Affiliated to Fudan University, Shanghai, 201799 PR China; 6 Wuxi Maternity and Child Health Care Hospital, Affiliated Women’s Hospital of Jiangnan University, Jiangsu, 214002 PR China; 7 Department of Pathology, Qingpu Branch of Zhongshan Hospital Affiliated to Fudan University, Shanghai, 201799 PR China

**Keywords:** Prostate cancer, MARCKSL1, Urine, Single-cell RNA sequencing, Liquid diagnosis

## Abstract

**Background:**

Prostate cancer (PCa) tissue biopsy has potential complications, and various methods guide biopsy decision. However, these methods have limitations. Urine-derived RNA shows promise for predicting and distinguishing PCa or clinically significant PCa (csPCa). The study aims to screen for prostate tumor cell-derived mRNAs in urine samples.

**Methods:**

Strand-specific capture RNA sequencing (RNA-seq) was performed on five paired tumor and adjacent normal prostate tissues from PCa patients, as well as ten urine samples derived from PCa patients and age-matched cancer-free healthy men. By integrating these transcriptomic data with single-cell RNA sequencing (scRNA-seq) datasets of three normal prostate tissues and 13 PCa tissues, we systematically screened and identified potential urinary biomarkers for PCa diagnosis. To further explore the diagnostic efficacy of the screened urinary biomarkers for csPCa, quantitative real-time polymerase chain reaction (qPCR) was conducted on 63 urine samples, including 33 samples from PCa patients and 30 samples from age-matched cancer-free healthy men. Additionally, in vitro gene knockdown experiments were implemented to preliminarily explore the biological functions of the identified urinary biomarkers.

**Results:**

Integrated analysis of strand-specific capture bulk RNA-seq and single-cell RNA-seq datasets identified urine-derived *MARCKSL1* as a highly specific candidate biomarker for PCa diagnosis. In vitro functional assays demonstrated that genetic knockdown of *MARCKSL1* robustly suppresses the migratory and invasive capacities of PCa cells. Furthermore, qPCR profiling verified that urine-derived *MARCKSL1* exhibits favorable diagnostic performance, allowing accurate stratification of patients with PCa and csPCa.

**Conclusions:**

Urine-derived *MARCKSL1* has the potential to facilitate pre-biopsy diagnosis and risk stratification of PCa and csPCa. Our findings may advance the development of non-invasive tools for pre-biopsy PCa risk evaluation and csPCa discrimination.

**Supplementary Information:**

The online version contains supplementary material available at https://doi.org/10.1186/s12894-026-02295-y.

## Introduction

Prostate cancer (PCa) presents a significant global health challenge. According to the latest estimates from the International Agency for Research on Cancer (IARC), it is ranked as the second most prevalent (14.2%) and fifth highest cause of mortality (7.3%) among men across 185 countries worldwide [1, 2]. The initial diagnosis of PCa primarily relies on histopathological examination of prostate tissue samples obtained through biopsy, utilizing the Gleason grading system as an evaluative criterion for pathological severity to guide treatment decisions [3–6]. The Gleason system, an architectural grading system ranging from 1 to 5 indicating the degree of differentiation (from well to poorly differentiated), facilitates a standardized risk assessment for patients with PCa presenting extraprostatic extension, biochemical recurrence, and overall survival [7–12]. The Gleason score (GS) is derived from both primary and secondary patterns within the Gleason grading system’s range of 2 to 10. It allows distinguishing between clinically significant PCa (csPCa; GS ≥ 7) associated with higher aggressiveness and worse prognosis, and indolent or insignificant PCa (isPCa; GS < 7) linked to lower aggressiveness and better prognosis. Consequently, this distinction leads to diverse management strategies and subsequent prognoses [13–15]. Unfortunately, GS remains unknown prior to prostate biopsy. Prostate biopsy procedures can render patients vulnerable to complications, including infection, hematuria, and pain. Furthermore, the risk of certain complications, particularly infection, escalates proportionally with the number of previous biopsies performed during active surveillance [16, 17]. Therefore, it is imperative to conduct noninvasive testing prior to prostate biopsy in order to accurately predict the risk of PCa and assess tumor aggressiveness, thereby eliminating unnecessary biopsies and reducing overdiagnosis [18].

The measurement of serum prostate-specific antigen (PSA) is a highly efficacious screening modality for the detection of PCa [19], commonly utilized in clinical practice to guide the decision on whether to proceed with a biopsy [20]. However, it should be noted that PSA is not exclusively specific to PCa, as it is also recognized as a common marker for other prostate diseases including benign prostatic hyperplasia (BPH), prostatitis, ejaculation-related trauma, and infection [21–23]. Moreover, the test inadequately distinguishes csPCa from isPCa [24, 25]. Therefore, its use as a primary screening test for PCa detection is challenging. In fact, the low specificity of serum PSA, uncertain benefits of PSA screening in reducing PCa-related deaths and potential harms associated with over-diagnosing indolent diseases have raised concerns about its utility. Consequently, most experts acknowledge that PSA testing increases the risk of over-diagnosis and subsequently exposes patients to potential morbidities related to overtreatment as well as anxiety that affects their quality of life [23]. Recent guidelines have recommended the routine use of multiparametric magnetic resonance imaging (mpMRI) in men with elevated PSA levels to inform the necessity for biopsy and reduce unnecessary procedures [26, 27]. The area under the receiver operator characteristic curves (AUCs) of mpMRI for predicting csPCa ranged from 0.79 to 0.84 [28]. However, mpMRI may pose risks for patients with internal fracture repair materials or pacemakers. Therefore, there is an urgent imperative to develop a safe, convenient, accurate, and robust test that can precisely predict PCa and effectively differentiate csPCa from isPCa prior to resorting to invasive biopsy procedures. Ideally, this diagnostic test should exhibit enhanced accuracy and noninvasiveness while utilizing easily obtainable samples such as urine.

The investigation of urine-derived biomarkers has been a prominent area of focus in experimental, clinical, and commercial research on PCa for several decades. Increasing studies suggest that urinary biomarkers can accurately predict PCa and effectively differentiate csPCa from isPCa aiding in decision-making regarding invasive biopsy procedures [29]. The proposed principle is that the continuous interaction between urine and tumor tissue facilitates the non-invasive acquisition of tumor information from exfoliated cells derived from neoplastic tissues. Testing urine-derived biomarkers may offer a convenient, safe, and cost-effective option for patients. It is worth mentioning firstly that the organism-specific homeostasis mechanism effectively eliminates a wide range of potential biomarkers, particularly during the early stages of tumorigenesis. However, due to the absence of such regulatory mechanisms, urine exhibits greater susceptibility to changes, especially in the initial phases of cancer development. Consequently, exploring urine-derived biomarkers holds promising potential for early-stage cancer detection even prior to employing tissue-based testing methods for tumor identification. Secondly, testing urine-derived biomarkers enables characterization of the entire tumor as opposed to specific tissue fragments assessed by prostate biopsy; thus it has the potential to identify significant locations within heterogeneous tumors that may be missed by biopsy procedures. Thirdly, sequential sampling of urine allows for progress monitoring, treatment modulation and early detection of recurrence which is more feasible compared to other methods.

Urine-derived biomarkers encompass DNA, RNA, and protein. mRNA biomarkers exhibit superior sensitivity and specificity compared to proteins while also being more cost-effective; furthermore, they enable physicians to make dynamic monitoring of disease occurrence and progression unlike DNA markers [30]. Hamid et al. developed an assay for the detection of *HOXC6* urinary mRNA and discovered that *HOXC6* was upregulated in urine samples from PCa patients compared to normal men [31]. In a study involving 1244 PCa patients, Tomlins et al. demonstrated that incorporating serum PSA with urine *TMPRSS2: ERG* and PCA3 scores outperformed serum PSA-based strategies for diagnosing PCa, highlighting the significance of these biomarkers in predicting advanced PCa (GS > 6) in needle biopsy with an AUC of 0.779 [32]. However, they still do not meet the criteria of being optimal benchmarks, necessitating the proposal of novel markers. Next generation sequencing (NGS)-based high-throughput detection experiments play a pivotal role in the screening of mRNA biomarkers; nevertheless, its application for urine mRNA screening has been limited by the low abundance and compromised integrity of mRNA in urine samples. To address this issue, we constructed RNA sequencing libraries following the SMARTer^®^ Stranded Total RNA-Seq Kit v2 protocol in this study to detect urine mRNA through high-throughput sequencing analysis. In comparison with poly-(A) enriched RNA sequencing libraries, our approach exhibits enhanced tolerance towards low mRNA integrity and abundance. We conducted a comprehensive assessment of the robustness and accessibility of urine RNA sequencing outcomes. Meanwhile, we integrated these results with single-cell RNA sequencing (scRNA-seq) data to identify a potential urine-derived biomarker, *MARCKSL1*, which originates from prostate tumor cells.

**Fig. 1:**
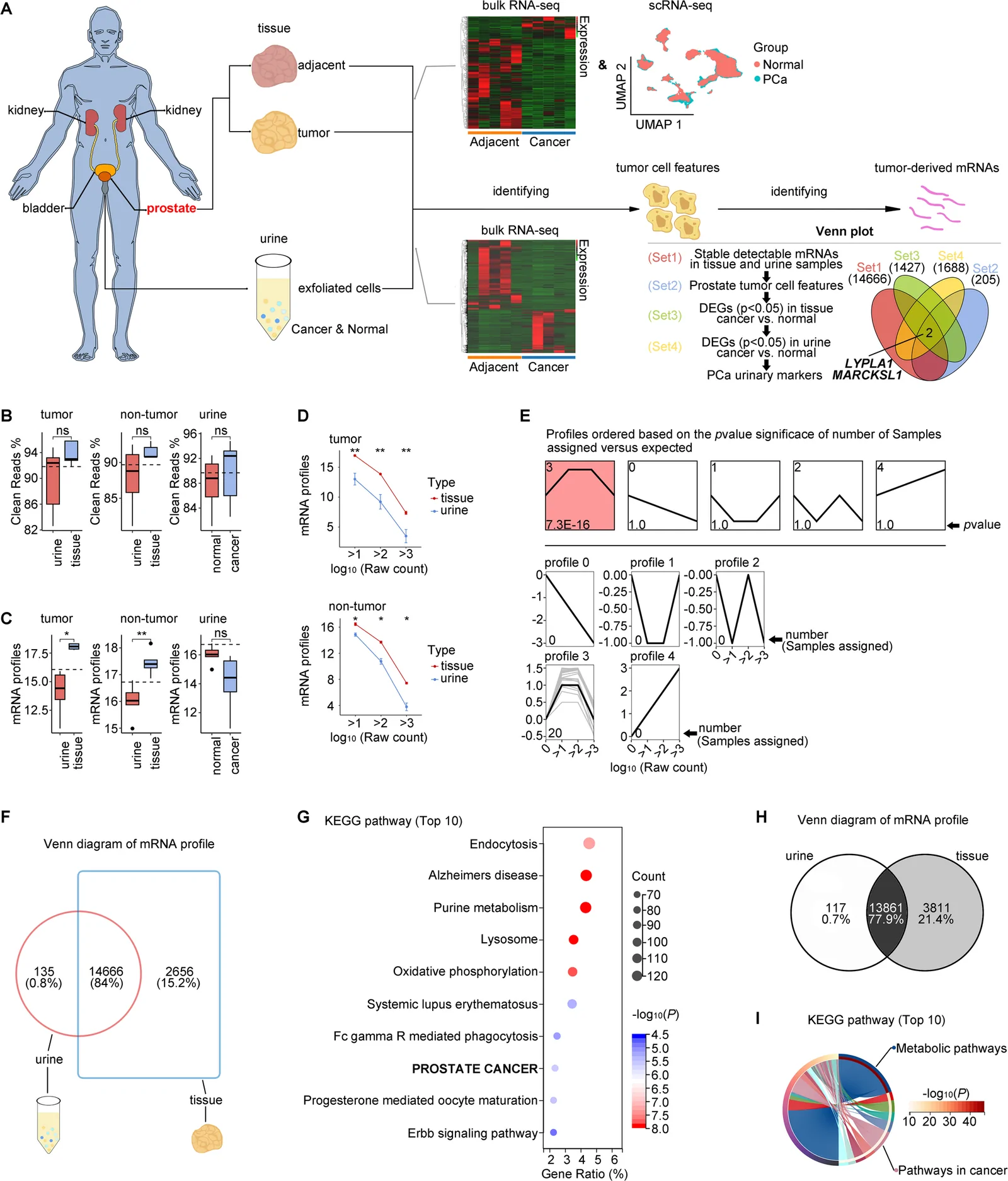
Quality evaluation of urine bulk RNA sequencing data. **A** Research flow chart drawn by Hiplot Pro (https://hiplot.com.cn/). **B** Box plot illustrating the comparison of the percentage of clean reads. **C** Box plot illustrating the comparison of the number of detectable mRNAs (raw count > 1). **D** Line chart illustrating the diversity of mRNA profiles across different levels of mRNA expression. **E** Trend analysis demonstrating the diversity of mRNA profiles between urine exfoliated cells and tissue samples at various levels of mRNA expression. **F** and **H** Venn diagram of mRNA profiles. **G** and (**I**) Top ten KEGG pathways exhibiting significant enrichment

Since being characterized as a primary substrate of protein kinase C, membrane-associated MARCKS has been established as a critical modulator of sophisticated cellular pathways [33]. As a MARCKS family paralog, MARCKSL1 shares highly similar physiological roles with MARCKS in cell proliferation, cell cycle control, migration, exocytosis, inflammation, and apoptosis [34, 35]. Abnormal MARCKSL1 expression has been detected in colorectal, lung and esophageal cancers [36–38], and previous work has indicated the diagnostic capacity of tissue MARCKSL1 for PCa [39, 40]. However, only a limited number of studies have validated its biological function, and no research has yet confirmed whether *MARCKSL1* in urine can serve as a marker for identifying PCa or csPCa. Accordingly, we assessed its biological functionality in prostate tumor cells and investigated the diagnostic efficacy of urine-derived *MARCKSL1* in cases of PCa and csPCa. The research procedure is illustrated in Fig. 1A. Our findings will not only offer a suitable and reliable high-throughput approach for screening urine-derived mRNA, but also provide innovative insights into non-invasive risk stratification diagnosis of PCa and the differentiation of csPCa prior to biopsy.

## Results

### Evaluating the robustness and accessibility of urine mRNA sequencing outcomes

The poly-(A) enrichment method is commonly employed for mRNA sequencing, and the quality of the resulting data heavily relies on the availability of high-quality RNA samples. Consequently, most commercial sequencing platforms require a minimum starting amount of 1µL total RNA and a RIN value exceeding 7 or even 8 to ensure reliable results. However, meeting these criteria poses challenges when working with urine-derived RNAs [41, 42]. Our findings revealed that the RIN scores of RNAs extracted from urine exfoliated cells were consistently below 7, while the total RNA yield ranged from 14.0ng to 400.8ng per 5mL urine (Figure S1A). Considering the inadequate quantity and quality of RNA in this particular case, which fell below the threshold required for mRNA sequencing through poly-(A) enrichment, we employed a strand-specific capture approach (SMARTer® Stranded Total RNA-Seq Kit v2) to address this limitation. By implementing this approach, we successfully achieved comparable output quality between RNA-seq utilizing urine-derived RNAs with low RIN scores and RNA-seq employing tissue-derived RNAs with high RIN scores. The proportions of clean reads derived from urine were consistent with those obtained from tissues in both tumor and non-tumor samples (*P* > 0.05). Moreover, no significant disparity was observed (*P* > 0.05) when comparing the proportion of clean reads obtained from urine tumor samples to those derived from urine samples of healthy individuals, indicating that the presence of PCa had minimal influence on the quality of urine RNA sequencing data, thus indicating its negligible impact (Fig. 1B). Furthermore, urine RNA-seq exhibited a comparable percentage of mapping to coding bases compared to tissue RNA-seq, indicating its capacity for effectively recalling coding bases (Figure S1B, Table S1).

Subsequently, we evaluated the number of detectable mRNAs (raw count > 1) in urine and tissue samples (Fig. 1C). Previous studies have reported a limited number of cell types in urine samples, including epithelial cells, leukocytes, and tumor cells [43]. Consequently, we observed a comparatively reduced diversity in the mRNA profiles of urine samples compared to tissues for both tumor (*P* = 0.011) and non-tumor samples (*P* = 0.0051). However, no significant difference was observed between tumor and non-tumor urine samples in terms of mRNA profile diversity (*P* > 0.05), indicating that the influence of tumor status on the variability of mRNA profiles in exfoliated cells from urine may be negligible. Furthermore, we examined the diversity of mRNA profiles in urine exfoliated cells across different levels of mRNA expression (Fig. 1D). Our findings demonstrated a gradual decrease in the number of detectable mRNAs in urine samples as mRNA expression levels increased, mirroring similar trends observed in tissue samples. The number of detectable mRNAs at each level of mRNA expression was significantly lower in urine compared to tissue samples. The trend analysis results revealed a significant concordance between the alterations in the number of detectable mRNAs and their corresponding expression levels observed in urine and tissue samples (*P* = 7.3E-16, Fig. 1E). These results suggest that urinary exfoliated cells retain stable tissue information across varying levels of mRNA expression.

A total of 14,801 and 17,322 stable detectable mRNAs were obtained from urine and tissue samples, respectively, with raw counts exceeding zero in over 50% of the analyzed samples. Among them, 84% (14,666) were found to be present in both sample types (Fig. 1f), exhibiting significant enrichment specifically within the prostate cancer pathway of the KEGG database (adj.*p* = 4.6E-05, Fig. 1G). Furthermore, a comparison between the number of stable detectable mRNAs in urine samples and their paired tumor tissue from the same PCa patient revealed that a majority of these mRNAs (77.9%) were present in both sample types (Fig. 1H). Additionally, they exhibited significant enrichment primarily in metabolic pathways (adj.*p* = 2.4E-45) and pathways related to cancer development (adj.*p* = 4.0E-10) according to the KEGG database analysis (Fig. 1I). These findings suggest that a substantial proportion of detectable mRNAs are present in urine, and these mRNAs also exist in tumor tissue samples, carrying tumor-related information.Consequently, they offer non-invasive valuable insights into PCa.

### Identifying the gene features of prostate tumor cells using scRNA-seq data

**Fig. 2:**
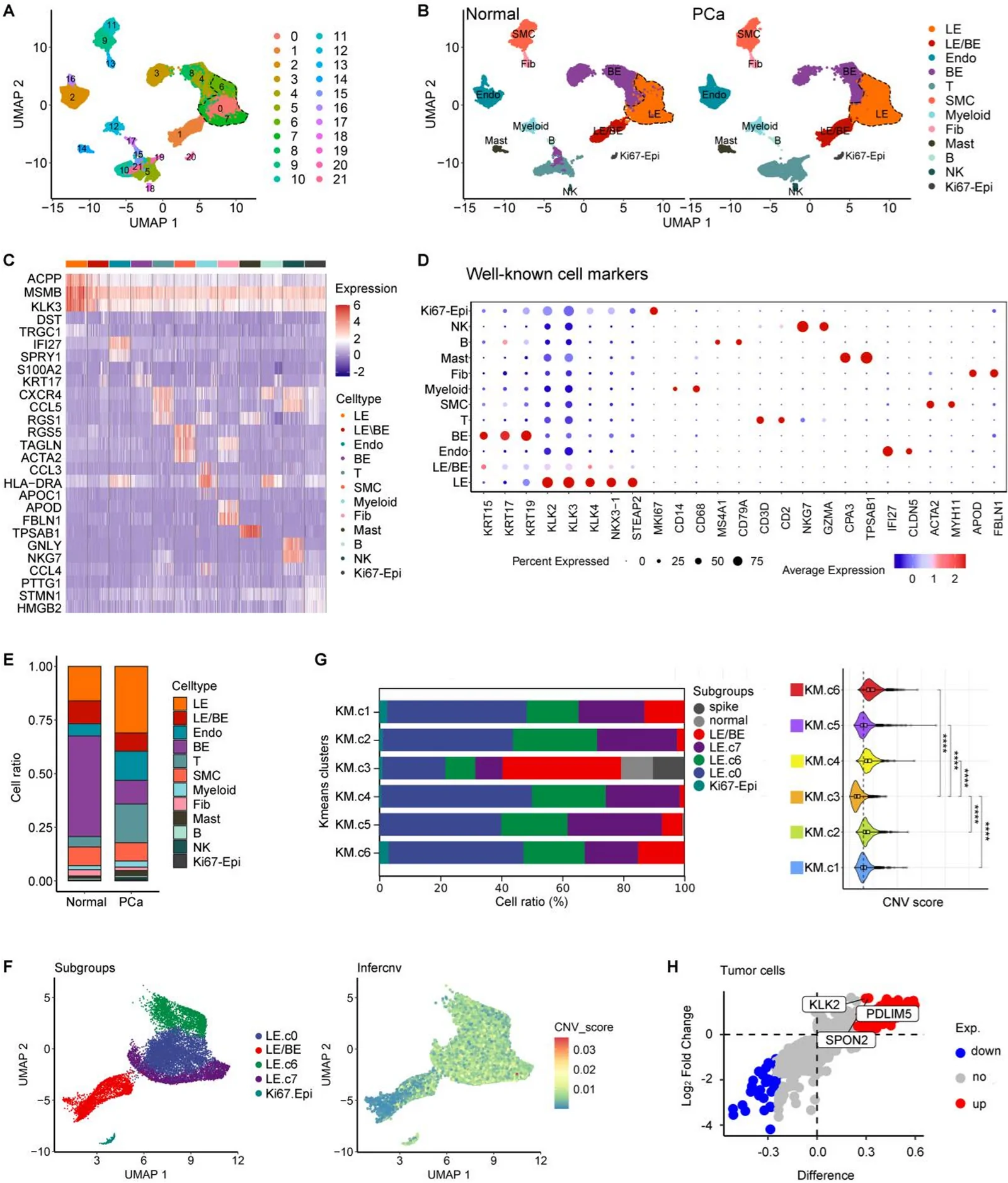
Identify the gene features of prostate tumor cells. **A** UMAP colored by clusters. **B** UMAP colored by celltypes. **C** Heatmap illustrating the top three DEGs within each celltype. **D** Dotplot of the expression levels of known cell markers in each cell type. **E** Ratio of cell types in each sample. **F** UMAP colored by epithelial cell subgroups and CNV scores. **G** Ratio of epithelial subtypes in each KMeans cluster clustered based on the CNV score. **H** DEGs of tumor cells versus other epithelial cells

To accurately discern the information pertaining to prostate tumor cells in urine, scRNA-seq data was utilized for the acquisition of the tumor cell genomic features. A total of twenty-two clusters were identified based on gene expression (Fig. 2A, Figure S2A-B). A total number of 52,968 cells were annotated as epithelial cells, stromal cells and immune cells based on established marker genes [44] (Fig. 2B-D, Figure S2C). *PTPRC* negative and *EPCAM* positive clusters were identified as epithelial cells, comprising luminal epithelial cells (LE) characterized by the expression of *KLK2*, *KLK3*, *KLK4*, *NKX3-1* and *STEAP2*; basal epithelial cells (BE) expressing *KRT15*, *KRT17*, and *KRT19*; Ki67-Epi cells expressing *MKI67*; and luminal-basal epithelial cells (LE/BE) characterized by the presence of both LE and BE markers. *PTPRC* positive clusters were identified as immune cells. Among them, the cluster exhibiting high-level expression of *CD3D* and *CD2* was designated as T cells; the cluster displaying high-level expression of *CD14* and *CD68* was classified as myeloid cells; the cluster demonstrating high-level expression of *NKG7* and *GZMA* represented natural killer (NK) cells. The remaining two *PTPRC* positive clusters were characterized as mast cells expressing *CPA3* and *TPSAB1*, and a population of B cells expressing *MS4A1* and *CD79A*. *PTPRC* and *EPCAM* negative clusters were identified as stromal cells, which consisted of endothelial cells (Endo) characterized by *CLDN5* and *IFI27* expression, fibroblasts (Fib) expressing *APOD* and *FBLN1*, and smooth muscle cells (SMC) expressing *ACTA2* and *MYH11*. The identification of the top three characteristic genes in each cell type provided further validation for the accurate annotation of these cells (Fig. 2C).

Prostate tumor cells mainly originate from LE cells. In this study, we observed a substantial augmentation in the population of LE cells within PCa tissues, in comparison to their presence in normal prostate tissues (Fig. 2E). Thus, to delve deeper into the gene features of prostate tumor cells, we performed subsequent analysis on three distinct LE clusters (LE.c0, LE.c6, and LE.c7), LE/BE cluster, and Ki67-Epi cluster. The inferCNV algorithm was utilized to compute the copy number variation (CNV) scores for individual cells, aiding in identifying tumor cells (Fig. 2F, Figure S2D). Our findings revealed that, utilizing an unsupervised KMeans clustering method, the cells were categorized into six distinct groups based on their CNV profiles. Predominantly, normal and spike normal cells were allocated to the KM.c3 group, which exhibited the lowest CNV scores among the six groups (Fig. 2G). Consequently, we inferred that all cells within the KM.c3 group were normal; conversely, the remaining groups comprised tumor cells. Subsequently, DEGs analysis was conducted to elucidate the gene features of these tumor cells. A total of 894 genes exhibited significant upregulation in tumor cells compared to normal epithelial cells (Fig. 2H). Among them, 205 genes were identified as characteristic features of prostate tumor cells, expressed in more than 70% of tumor cells and less than 50% of other normal epithelial cells, with significant enrichment observed within the Prostate cancer pathway of the KEGG database (adj.*p* = 1.96E-02; Figure S3, Table S2).

### Integrating bulk RNA-seq and scRNA-seq data identifying of tumor cell derived biomarkers in urine

**Fig. 3:**
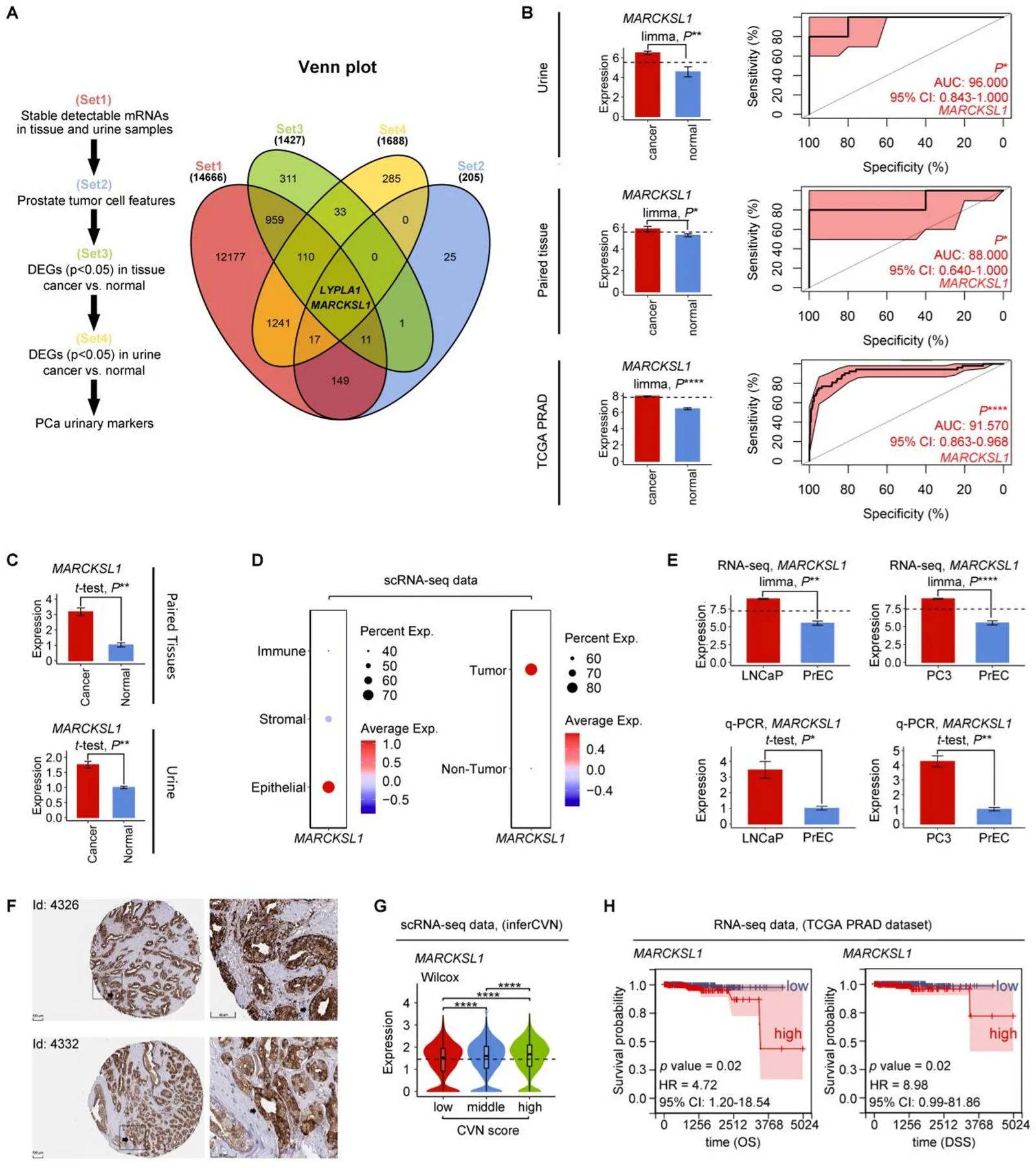
Identification of urinary tumor cell biomarkers. **A** Strategic diagram for the identification of the urinary exfoliated prostate tumor cell biomarkers. **B** Bar plot illustrating the comparison of the expression levels of *MARCKSL1* in PCa and normal urine and tissue samples (RNA-seq data); receiver operating characteristic curve (ROC) plots demonstrating the diagnostic accuracy of *MARCKSL1* in both urine and tissue samples for PCa (RNA-seq data). **C** Bar plot illustrating the comparison of the expression level of *MARCKSL1* in PCa and normal urine and paired tissue samples (qPCR data). **D** Dotplot of the expression level of *MARCKSL1* in each cell type (scRNA-seq data). **E** Bar plot illustrating the comparison of the expression level of *MARCKSL1* in prostate tumor and normal epithelial cell lines (RNA-seq and qPCR data). **F** IHC data demonstrating the expression of MARCKSL1 in prostate tissues obtained from patients with PCa. **G** Violin plots illustrating the comparison of the expression level of *MARCKSL1* in prostate tumor cells across different ranges of CNV scores: low (0 - Quartile 1), middle (Quartile 1 - Quartile 3), and high (Quartile 3–100%) scores (scRNA-seq data). **H** Kaplan-Meier (KM) curves illustrating the prognostic value of *MARCKSL1* for OS and DSS in the TCGA PCa cohort

Subsequently, we conducted a comprehensive screening and identification for specific tumor cell derived biomarkers in urine. Initially, we conducted DEGs analysis on urine sequencing data from cancer and normal samples, as well as paired prostate tumor and adjacent normal tissue sequencing data. A total of 1688 genes exhibited significant differential expression in urine exfoliated cells from patients with PCa compared to those in urine exfoliated cells from healthy men, while a total of 1427 genes showed significant differential expression in the paired prostate tumor tissues compared to the adjacent normal tissues of the PCa patients. Then, the intersection analysis was conducted using the aforementioned two sets of DEGs and gene sets, encompassing a total of 14,666 stable detectable mRNAs in both urine exfoliated cells and prostate tissues (Fig. 3A). Furthermore, an additional set comprising 205 signatures specific to prostate tumor cells was also integrated into the analysis. Consequently, *LYPLA1* and *MARCKSL1* were identified across all four gene sets. Notably, the upregulation of *MARCKSL1* demonstrated consistent stability in both PCa urine samples and tumor tissue samples when compared to normal urine samples and adjacent normal tissue samples (Fig. 3B, Figure S4A-B). These findings were further validated through qPCR analysis, which supported our sequencing results (Fig. 3C). Consequently, we subsequently conducted a series of comprehensive analyses to elucidate the potential of urinary *MARCKSL1* as an informative marker for prostate tumor cells.

By utilizing our urine samples and integrating them with paired prostate tumor and adjacent normal tissue sequencing data, as well as the Cancer Genome Atlas (TCGA) PCa cohort sequencing data, we have observed that *MARCKSL1* exhibited exceptional diagnostic accuracy for PCa, substantiated by the impressive AUCs of 96.00 (95%CI: 0.843-1.000), 88.00 (95%CI: 0.640-1.000), and 91.57 (95%CI: 0.863–0.968) (Fig. 3B). The scRNA-seq data revealed predominant expression of *MARCKSL1* in prostate epithelial cells rather than immune or stromal cells; specifically within the epithelial cell population, it is significantly upregulated in tumor cells compared to non-tumor cells (Fig. 3D). Transcriptomic profiling across seven PCa cell lines (H660, LNCaP, VCaP, C4-2, 22RV1, PC3, DU145) confirmed ubiquitous MARCKSL1 transcription (Figure S5). Consistent upregulation of *MARCKSL1* was also detected in representative PCa cell lines (LNCaP and PC3) compared with normal prostate epithelial cells (PrEC), a finding further validated via qPCR quantification (Fig. 3E). Immunohistochemistry (IHC) staining corroborated that MARCKSL1 protein was primarily enriched in prostatic glandular epithelial cells (Fig. 3F and Figure S6). Notably, PCa tissues exhibited substantially higher proportions of moderate and strong MARCKSL1 protein positivity than normal prostate tissues (Figure S7). Besides, to eliminate transcriptional interference from other urogenital organs, we conducted cross-tissue expression analysis using public transcriptome resources from the Genotype-Tissue Expression (GTEx) Project and the Functional Annotation of the Mammalian Genome 5 (FANTOM5) Project. *MARCKSL1* transcript abundance was markedly higher in prostate tissue than in renal and bladder tissues (Figure S8). Collectively, these lines of evidence support *MARCKSL1* as a cell-type-specific marker for malignant prostate epithelial cells and lay a solid basis for its diagnostic application in PCa. Furthermore, our analysis of scRNA-seq data revealed a significant correlation between the expression level of *MARCKSL1* and tumor cell malignancy (Fig. 3G). For prognostic evaluation, TCGA PCa patients were stratified into high-*MARCKSL1* (3rd quartile to maximum) and low-*MARCKSL1* (minimum to 1st quartile) subgroups. Patients with elevated *MARCKSL1* expression presented significantly worse overall survival (OS) and disease-specific survival (DSS) outcomes (Fig. 3H). Taken together, our results highlight the dual translational value of *MARCKSL1*: it can reflect the malignant degree of prostate lesions and serve as a molecular indicator for prognostic risk stratification.

### *MARCKSL1* affecting prostate tumor cellular migration and invasion

**Fig. 4:**
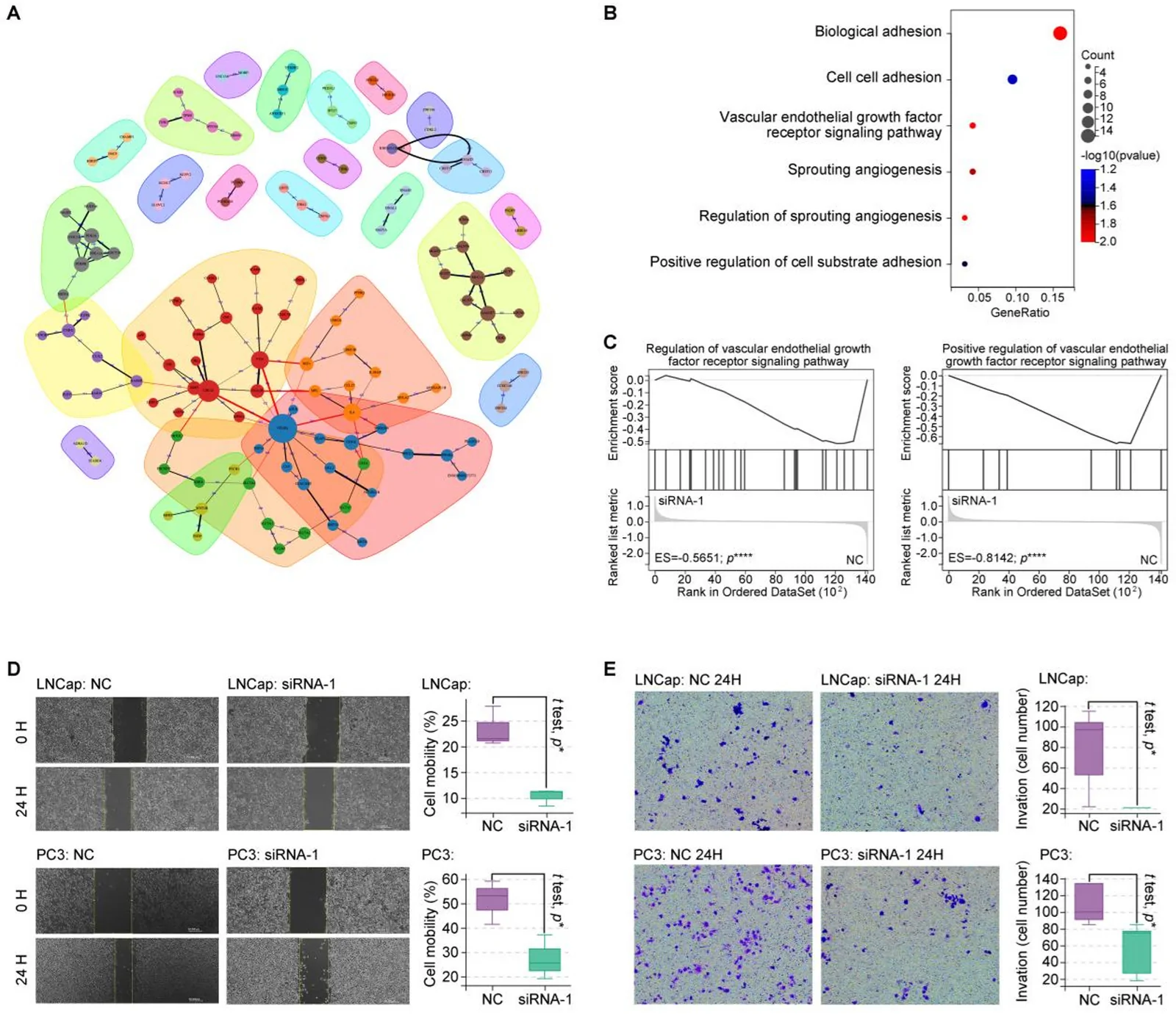
*MARCKSL1 *affecting prostate tumor cellular migration and invasion. **A** PPI network of downregulated genes in *MARCKSL1*-knockdown PC3 cells. **B** GOBPs exhibiting significant enrichment. **C** GSEA plots interpreting changes in the expression levels of the regulation of vascular endothelial growth factor receptor signaling pathway and the positive regulation of vascular endothelial growth factor receptor signaling pathway across knockdown cells and corresponding controls. **D** Representative images of wound-healing assays using PC3 and LNCap cells, showing cell motility after knockdown of *MARCKSL1* (left panels). Scale bar, 50 μm. Box plots illustrating the comparison of cell migration distances (right panels). **E** Representative images of invasion assays using PC3 and LNCap cells (left panels), showing cell invasion after knockdown of *MARCKSL1*. Magnification, 100X. Box plots illustrating the comparison of the invaded cell counts (right panels)

To gain deeper insights into the diagnostic role of *MARCKSL1* in PCa, we conducted a comprehensive investigation into its biological functionality. Transient transfection with four small interfering RNAs (siRNAs) (siRNA-1, siRNA-2, siRNA-3, and siRNA-4) resulted in a significant downregulation of *MARCKSL1* expression levels in PC3 and LNCap cells compared to their corresponding control prostate tumor cells which were treated with negative control RNA oligonucleotides (NC) (Figure S9A-B). Notably, siRNA-1 exhibited pronounced effects, prompting subsequent RNA sequencing analysis of total RNAs isolated from PC3 cells treated with siRNA-1 and NC, respectively. The results demonstrated a significant downregulation of cell migration-related genes, such as *CDC42* and *VEGFA*, which were identified as the hub molecules in the protein-protein interaction (PPI) network within the *MARCKSL1* knockdown PC3 cells compared to their corresponding controls (Fig. 4A). Moreover, the over-representation analysis (ORA) results showed a significant enrichment of downregulated genes involved in Gene Ontology Biological processes (GOBPs), such as cell-cell adhesion and the vascular endothelial growth factor receptor signaling pathway - crucial pathways for cellular migration and invasion - within the *MARCKSL1* knockdown PC3 cells (Fig. 4B). Additionally, gene set enrichment analysis (GSEA) results revealed a substantial downregulation of both regulation of vascular endothelial growth factor receptor signaling pathway and positive regulation of vascular endothelial growth factor receptor signaling pathway within the *MARCKSL1* knockdown PC3 cells (Fig. 4C). These findings underscore the potential role of *MARCKSL1* in mediating prostate tumor cell migration.

Subsequently, wound healing migration assays were performed to validate our findings. Our data revealed a significant reduction in the 24-hour cell migration distances of *MARCKSL1* knockdown PC3 and LNCap cells compared to their respective controls, indicating impaired migrational ability (Fig. 4D, Figure S9C). Furthermore, transwell assays were performed to investigate the impact of *MARCKSL1* on the invasive potential of prostate tumor cells. The results demonstrated a significantly decreased migratory capacity of *MARCKSL1* knockdown PC3 and LNCap cells compared to control cells after a 24-hour incubation period, as evidenced by a reduced number of cells traversing from the upper surface of the matrigel-coated chamber to reach the lower surface (Fig. 4E, Figure S9D). Overall, these findings underscore the potential involvement of *MARCKSL1* in facilitating migration and invasion of prostate tumor cells, thereby providing theoretical support for *MARCKSL1* as a prognostic indicator for poor prognosis in PCa.

### Evaluating the diagnostic potential of urinary exfoliated cell *MARCKSL1* for PCa

**Fig. 5:**
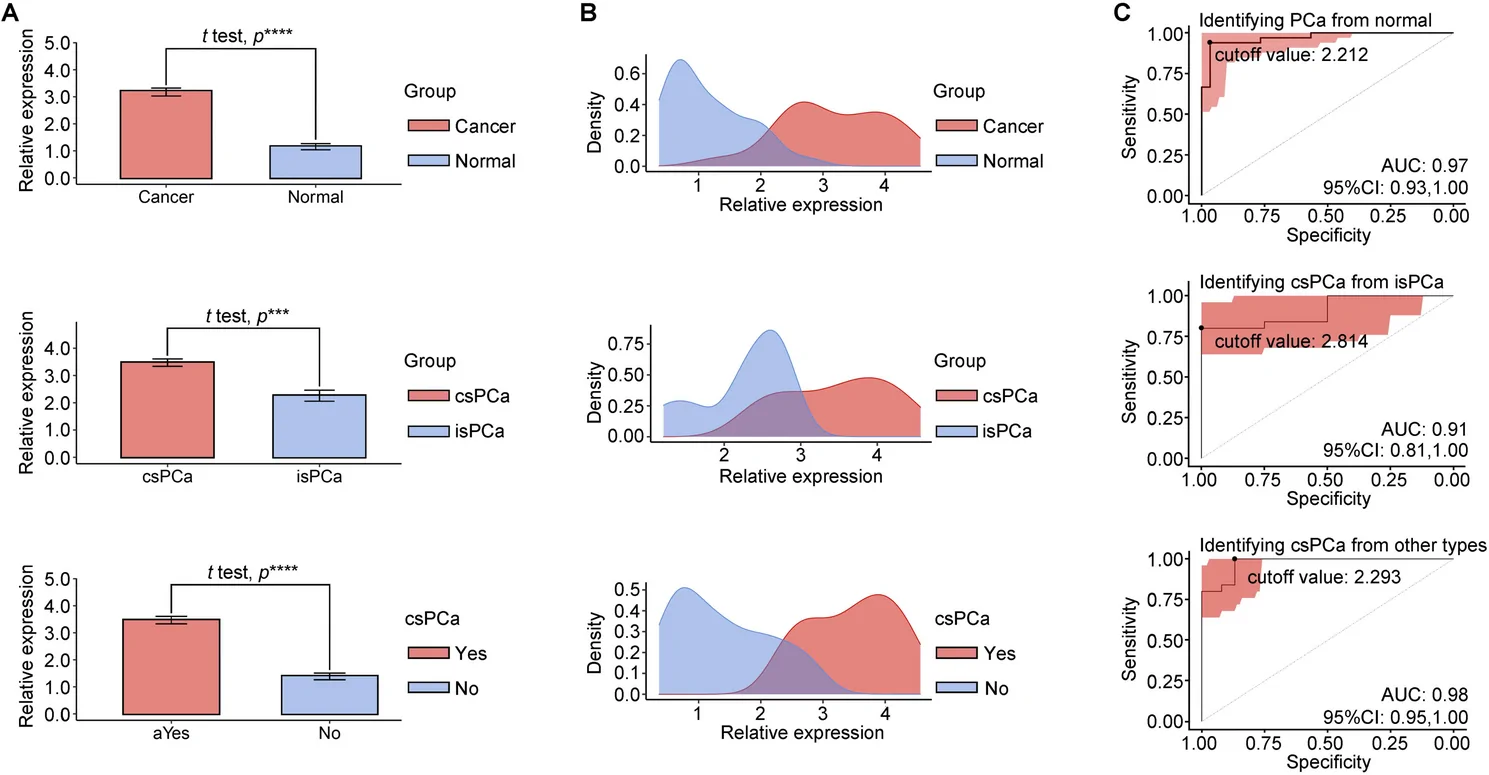
Evaluating the diagnostic potential of urinary exfoliated cell *MARCKSL1 *for PCa and csPCa. **A** Bar plot illustrating the expression levels of *MARCKSL1* in urinary exfoliated cells (qPCR data). **B** Density histograms illustrating the expression levels of *MARCKSL1* in urinary exfoliated cells (qPCR data) (**C**) ROC plots demonstrating the diagnostic accuracy of urinary exfoliated cell *MARCKSL1* for PCa and csPCa (qPCR data)

We conducted qPCR analysis on a total of 33 urinary exfoliated cell samples obtained from patients with PCa, as well as 30 urinary exfoliated cell samples collected from healthy men, in order to assess the diagnostic potential of urinary exfoliated cell *MARCKSL1* for PCa. Our results demonstrated significantly elevated expression levels of *MARCKSL1* in patients with PCa compared to healthy controls (Fig. 5A). Moreover, among these 33 PCa patients, 25 were classified as csPCa and exhibited markedly higher expression levels of *MARCKSL1* not only compared to isPCa patients but also healthy controls. The present findings validate the strong correlation between urinary exfoliated cell-associated *MARCKSL1* and PCa, particularly highlighting its pronounced association with csPCa when compared to serum PSA levels (Figure S10A-B). The density analysis revealed that the initial peak of *MARCKSL1* expression in the healthy controls was predominantly localized within the 0–1 units region, whereas patients with PCa and csPCa exhibited a delayed onset of urine *MARCKSL1* expression after 2 units (Fig. 5B). It is noteworthy that the peak expression of *MARCKSL1* in urinary exfoliated cell samples from patients with csPCa occurred at 3, while there was minimal overlap in the expression peak of *MARCKSL1* between the healthy controls and patients with isPCa. Furthermore, a significant positive correlation was observed between the levels of urinary exfoliated cell *MARCKSL1* and GS (Figure S10C). These findings suggest that urinary exfoliated cell *MARCKSL1* has the potential to distinguish PCa, particularly csPCa, from both healthy controls and patients with isPCa.

Receiver operating characteristic curve (ROC) analyses were conducted. The results revealed that urinary exfoliated cell *MARCKSL1* had an AUC value of 0.97 for distinguishing between patients with PCa and healthy individuals (Fig. 5C). When the cut-off value was set at 2.212, the urinary exfoliated cell *MARCKSL1* showed a sensitivity of 0.939 and a specificity of 0.967 in discriminating PCa from healthy controls, resulting in a diagnostic accuracy of 0.952 and a Youden index of 0.906 (Table 1). A systematic review and meta-analysis study involving 14,489 participants reported that the sensitivity, specificity, and AUC value of serum PSA for PCa were 0.93 (95%CI: 0.88–0.96), 0.20 (95%CI: 0.12–0.33), and 0.72 (95%CI: 0.68–0.76) [45], suggesting that urinary exfoliated cell *MARCKSL1* may have a superior discriminatory ability to distinguish PCa patients from healthy individuals compared to serum PSA. Another ROC analysis was conducted, and it was found that urinary exfoliated cell *MARCKSL1* had an AUC value of 0.91 for differentiating csPCA from isPCA (Fig. 5C). With a cut-off value set at 2.814, urinary exfoliated cell *MARCKSL1* demonstrated a sensitivity of 0.800 and specificity of 1.000, yielding a diagnostic accuracy of 0.848 and a Youden index of 0.800 respectively (Table 1, Figure S10D). It showed notably superior performance in diagnosing csPCA compared to serum PSA (Figure S10B). When the cut-off value of serum PSA was set at 19.35 mg/ml, it achieved optimal sensitivity and specificity for diagnosing csPCa. Serum PSA yielded a sensitivity of 0.65 and a specificity of 0.846, resulting in a diagnostic accuracy of 0.727 and a Youden index of 0.496. Moreover, a third ROC analysis was conducted, and the results showed that urinary exfoliated cell *MARCKSL*1 had an AUC value of 0.98 for discerning csPCA from both isPCA patients and healthy individuals (Fig. 5C). When the cut-off value was set at 2.229, the urinary exfoliated cell *MARCKSL1* displayed a sensitivity and specificity of 1.000 and 0.816 respectively, leading to a diagnostic accuracy of 0.889 along with a Youden index value reaching up to 0.816 (Table 1, Figure S10D). These findings indicate that urinary exfoliated cell *MARCKSL1* exhibits better diagnostic potency than serum PSA and possesses remarkable potential as an effective biomarker for accurate identification not only between PCa cases and healthy individuals but also specifically for distinguishing csPCa cases.

**Table 1:** Diagnostic potential of urinary exfoliated cell *MARCKSL1* for PCa and csPCa

Description	Sensitivity	Specificity	Diagnostic accuracy, AC	Youden index, YI
Discriminating PCa from healthy controls^a^	0.939	0.967	0.952	0.906
Discriminating csPCa from isPCa^b^	0.800	1.000	0.848	0.800
Discriminating csPCa from both isPCa and healthy controls^c^	1.000	0.816	0.889	0.816

^a^cut-off value was set as 2.212

^b^cut-off value was set as 2.814

^c^cut-off value was set as 2.229

## Discussion

Histopathological verification through systematic tissue biopsy remains the gold standard for establishing a definitive diagnosis of PCa. However, each prostate biopsy carries the potential risk of complications, and the likelihood of specific complications increases with the cumulative number of prior biopsies conducted during active surveillance [16, 17]. Although various approaches, such as serum PSA testing, digital rectal examination (DRE), and magnetic resonance imaging (MRI), have been employed to aid in decision-making regarding the necessity of conducting a prostate biopsy, all these methods possess inherent limitations. The diagnostic specificity of PSA for detecting PCa is suboptimal, and its accuracy in identifying csPCa is limited. In this study, we observed an AUC value of 0.727 for serum PSA in distinguishing csPCa from isPCa cases. Therefore, relying on PSA as a primary screening tool for PCa detection presents significant challenges. DRE is often perceived as a discomforting experience by patients [46]. MRI has emerged as a crucial tool in the detection and evaluation of PCa, highlighting the significance of mpMRI in guiding targeted biopsies while minimizing unnecessary sampling [47]. However, it has certain limitations including potential false-positive and false-negative results as well as challenges in accurately identifying csPCa. Furthermore, interpretation of mpMRI images requires expertise, leading to inherent variability in outcomes. Additionally, its widespread availability may be constrained, particularly in resource-limited settings, while cost considerations also pose challenges. Hence there is an urgent need to develop a reliable, convenient, precise, and robust test that can accurately predict the presence of PCa and effectively differentiate csPCa before resorting to invasive biopsy procedures.

Urine-derived biomarkers testing offers a more convenient and cost-effective alternative for patients with PCa. It allows for comprehensive characterization of the entire tumor, in contrast to tissue biopsies that only evaluate specific fragments of heterogeneous tumors and may potentially overlook approximately 21%-28% of PCa cases [48]. Furthermore, the sequential sampling of urine-derived biomarkers testing enables feasible progress monitoring, treatment modulation, and early detection of recurrence. The mRNA biomarkers, as one of the urine-derived biomarkers, exhibit superior sensitivity and specificity compared to proteins, while also being more cost-effective. Additionally, they offer a more dynamic insight into cellular regulation and state than DNA markers [30], thus garnering increased attention for the investigation of urine-derived mRNA screening. However, two factors may hinder the progress of such investigations. The first factor pertains to identifying the most suitable and robust high-throughput method for screening urine-derived mRNA in research studies. The mRNA extracted from urine exhibits low abundance and compromised integrity. In this study, the RIN scores of RNAs extracted from urine exfoliated cells consistently remained below 7, while the total RNA yield ranged from 14.0ng to 400.8ng per 5mL urine. Both their abundance and integrity were insufficient for mRNA sequencing using the poly-(A) enrichment method. Therefore, in this study, we employed a high-throughput detection method based on strand-specific capture using the SMARTer^®^ Stranded Total RNA-seq Kit v2. The method exhibits exceptional tolerance towards the quality of total RNA samples, obviating the need for supplementary rRNA removal techniques or kits and facilitating a reduction in RNA loss during manipulation. By comparing the mRNA sequencing results of tumor tissue, adjacent normal tissue, and urine samples obtained from the same PCa patient, we observed that this method successfully achieved comparable output quality between RNA-seq utilizing urine-derived RNAs with low RIN scores and RNA-seq employing tissue-derived RNAs with high RIN scores. The ability to accurately recall encoded bases was similar between them as well. Meanwhile, urine-derived mRNAs consistently preserved tumor-related information across various expression levels present in tumor tissue samples. Therefore, we propose that the strand-specific capture method (SMARTer^®^ Stranded Total RNA-seq Kit v2) is a suitable and reliable high-throughput approach for urine-derived mRNA screening. Furthermore, we also observed a significant enrichment of mRNAs shared by prostate tumor tissue and urine within the prostate cancer pathway according to KEGG database analysis results. These findings support that there is a substantial presence of detectable mRNA in urine samples corresponding to tumor-related information which offers valuable support for non-invasive access to insights for PCa patients.

The second factor concerns the identification of tumor cell-derived mRNAs in urine, specifically addressing whether the screened urine-derived mRNAs possess sufficient specificity and sensitivity for tumor recognition. To comprehensively address this issue, we propose an integrated approach that combines scRNA-seq data with bulk sequencing data from both tissue and urine samples. Initially, scRNA-seq data from PCa tissue was employed to identify a signature set of 205 genes specific to prostate tumor cells. Subsequently, through sequencing analysis of tumor tissues, adjacent normal tissues, and urine samples obtained from the same PCa patients, we identified 1427 significantly differentially expressed mRNAs in both PCa patient and healthy individual urine samples as well as paired prostate tumor tissues and adjacent normal tissues. Finally, employing intersection analysis using the aforementioned gene sets successfully pinpointed two candidate mRNAs likely derived from urinary prostate tumor cells: *LYPLA1* and *MARCKSL1*. Notably, when compared to normal urine samples and adjacent normal tissue counterparts, *MARCKSL1* consistently exhibited stable upregulated expression levels in both PCa urine samples and corresponding tumor tissue specimens. Besides, cell RNA-seq data indicated that *MARCKSL1* was expressed in both androgen-sensitive and androgen-insensitive PCa cells, and its expression levels were higher than those in normal prostate epithelial cells. Moreover, tissue-derived *MARCKSL1* expression was found to be closely associated with malignant progression of tumor cells as well as OS and DSS among PCa patients. These findings underscore the potential utility of *MARCKSL1* not only for diagnosing PCa but also for indicating tumor malignancy and predicting prognosis.

The MARCKSL1 protein, a member of the myristoylated alanine-rich C kinase substrates (MARCKS) family, is characterized by its myristoylation and high content of alanine residues [34]. Its primary function involves phosphorylating and bundling actin filaments to enhance filopodia dynamics [49]. Previous studies have reported that MARCKSL1 plays a crucial role in coordinating cytoskeletal organization at cell-cell and cell-substrate contacts in epithelial cells, suggesting its potential relevance to cellular motility [50]. Several studies have consistently demonstrated the pivotal role of MARCKSL1 in promoting the migration and invasion of tumor cells [49, 51]. Our present investigation corroborates these findings by revealing a significant impairment in migration and invasion of prostate tumor cells following knockdown of *MARCKSL1*. Moreover, through ORA and GSEA analysis of RNA-seq data subsequent to knockdown of *MARCKSL1*, we identified a pronounced downregulation of pathways associated with cell-cell adhesion and the vascular endothelial growth factor receptor signaling pathway - both crucial for facilitating cellular migration and invasion. These findings underscore the potential involvement of *MARCKSL1* in facilitating migration and invasion of prostate tumor cells, thereby providing theoretical support for considering *MARCKSL1* as a prognostic indicator for poor prognosis in PCa. Although several studies have proposed the diagnostic potential of tissue-derived *MARCKSL1* in PCa [39, 40], there is currently insufficient evidence to support the utility of urine-derived *MARCKSL1* for identifying PCa or csPCa. In this study, our findings confirmed that urinary exfoliated cell *MARCKSL1* originates from tumor, and by conducting qPCR analysis on 33 urinary exfoliated cell samples obtained from patients with PCa as well as 30 urinary exfoliated cell samples collected from healthy controls, we propose that urinary exfoliated cell *MARCKSL1* exhibits robust diagnostic potential for accurately identifying PCa patients, particularly in distinguishing those with csPCa from both those with isPCa and normal individuals, achieving a sensitivity and specificity exceeding 80%.

Although our research has yielded meaningful results, several inherent limitations must be acknowledged. First, the concentration of exfoliated cells in urine fluctuates substantially due to variables including water intake and micturition intervals. Such variability may interfere with the determination of diagnostic thresholds for PCa and csPCa assays, as well as compromise the sufficiency and rationality of urine sampling volumes. This hurdle complicates the clinical translation of the present work and other urine-based biomarker studies, indicating that further procedural optimization is required to address this gap. Second, this study relied on single-center patient recruitment with a limited cohort size. Additional multicenter trials should be carried out to validate our findings and facilitate their clinical translation. 

## Conclusions

In conclusion, our findings demonstrate that the implementation of a high-throughput approach for urine-derived mRNA screening utilizing the strand-specific capture technique (SMARTer^®^ Stranded Total RNA-seq Kit v2) is both suitable and reliable. This method enables stable acquisition of detectable mRNA profiles carrying tumor-related information from urine samples. Furthermore, through an integrated analysis combining scRNA-seq with tissue and urine bulk sequencing data, urinary exfoliated cell *MARCKSL1* has been first identified as a robust diagnostic biomarker for accurately distinguishing patients with PCa, particularly in differentiating between csPCa patients, isPCa patients, and normal individuals. Our findings provide innovative insights into non-invasive risk stratification diagnosis of PCa and the differentiation of csPCa prior to biopsy.

## Methods

### Sample collection and preparation

This study was approved by the Ethical Committee of QingPu Branch of Zhongshan Hospital (Qingyi2020-19), and all participants provided informed consent. For research purposes, samples were collected from January 11, 2021, to January 11, 2023. Urine samples were collected from 63 patients who visited QingPu Branch of Zhongshan Hospital. Among them, 33 patients without a history of cancer underwent prostate biopsy due to clinical suspicion of PCa, and a first diagnosis of PCa was histopathologically established based on the analysis of biopsy tissue. Urine samples were collected from 33 PCa patients prior to prostate biopsy. Thirty age-matched healthy men were free of any malignant diseases and had no personal history of systemic malignancy. They had no clinical indications for performing prostate biopsy, and PCa was clinically excluded based on comprehensive screening. All 63 urine samples were collected as clean-catch midstream urine specimens and stored at 4 °C prior to centrifugation for isolation of exfoliated urinary cells. Additionally, a total of five pairs of PCa tissue and adjacent normal tissue were procured from the surgical site. Table S3 contains relevant clinical information pertaining to PCa patients.

### Data download

The datasets used in this study were acquired from the TCGA database, and the NCBI Gene Expression Omnibus (GEO) database including GSE70466, GSE210205, GSE172357 (GSM5252457, GSM5252459, and GSM5252461), GSE141445 (10 samples of GSM4203181), GSE157703 (GSM4773521 and GSM4773522), as well as GSE176031 (GSM5353236 and GSM5353237). The IHC data of normal and tumor prostate tissues and scRNA-seq data of prostate tissues were obtained from the Human Protein Atlas database (https://www.proteinatlas.org) [52–54]. The cross-tissue data for RNA expression was obtained through the GTEx and the FANTOM5 project. The sequencing datasets of seven wild-type prostate cell lines, namely H660, LNCaP, VCaP, C4-2, 22RV1, PC3, and DU145 cells, were downloaded from CTPC (https://pcatools.shinyapps.io/CTPC_V2/) [55].

### RNA extraction

Total RNA was extracted using TRIzol (Invitrogen, CA), followed by assessment of concentration, purity and integrity using NanoDrop 2000 spectrophotometer (Thermo Scientific, USA) and the Agilent 2100 Bioanalyzer RNA Pico kit (Agilent Technologies, Santa Clara, CA).

### Bulk RNA-seq and data processing

The sequencing libraries were prepared from total RNAs obtained from ten urine exfoliated cells and five paired tumor tissues and adjacent normal tissues following the SMARTer^®^ Stranded Total RNA-Seq Kit v2 protocol (Takara Bio Inc., JPN). Additionally, poly-(A) enriched RNA sequencing libraries were prepared from total RNAs obtained from cell lines. The Illumina platform was used for a 150 bp paired-end run to sequence the libraries. TopHat2.0.1 was employed to map the reads to the hg38 human genome, and mapped reads were filtered based on their mapping quality. GSE70466 and GSE210205 datasets retrieved from the GEO database were utilized to access the mRNA profiles of cell lines. All gene expression matrices were log-transformed to log2(Fragments Per Kilobase Million (FPKM) + 1). Multiple matrices were merged using the inSilicoMerging R software package, followed by batch effect removal through the Empirical Bayes method (ComBat) [56]. Differential expression genes (DEGs) analysis was performed using the Limma R package, while gene set enrichment analysis was conducted with the clusterProfiler R package. Trend analysis was carried out using STEM-Short Time-series Expression Miner (http://www.cs.cmu.edu/~jernst/stem).

### Single-cell RNA-sequencing data analysis

The published scRNA-seq datasets (GSE172357, GSE141445, GSE157703, and GSE176031) were obtained from the NCBI GEO database. Among these datasets, three samples were obtained from normal prostate tissues, and thirteen samples were obtained from PCa tissues. All datasets were loaded into the R package Seurat (Version 4.4.0) for subsequent analysis, and low-quality cells were excluded. Subsequent processes, including data normalization, integration (dims = 1:50), batch effect removal, cell cycle assessment, and cell annotation, followed the scRNA-seq analysis protocol described in our previous studies [44, 57]. The general information of these published scRNA-seq datasets was shown in Table S4.

### IHC staining

All images were analyzed and quantified using ImageJ software.

### Cell lines and culture conditions

The established PC-3/LNCaP prostate cancer cell lines and the PrEC prostate epithelial cell line were utilized in this study. PC-3/LNCaP cells and RWPE1 cells were cultured at 37 °C and 5% CO2 in RPMI-1640 medium and Prostate Epithelial Cell Medium supplemented with 100 U/mL penicillin, 100 µg/mL streptomycin, 1 mM sodium pyruvate, and 10% foetal bovine serum (FBS). All reagents were purchased from Gibco (USA), Sigma-Aldrich (USA), or ScienCell (USA).

### Transient transfection assay

siRNA oligonucleotides targeting *MARCKSL1* (termed as siRNA-1, siRNA-2, siRNA-3, and siRNA-4) and negative control RNA oligonucleotides (termed NC) were designed and synthesized by GenePharma Biotech (CHN). The sequence of siRNA is shown in Table S5. The prostate tumor cells were transfected with siRNA using Lipofectamine™2000 (Thermo Scientific, USA) following the manufacturer’s instructions.

### Wound healing migration assays

The cells were seeded into 6-well plates and cultured until they reached 90% confluency. A sterile 200 µL pipette tip was utilized to generate a linear scratch on the cellular monolayer. Subsequently, the cells were thoroughly washed to eliminate any cellular debris and incubated in serum-free medium at 37 °C. The process of wound closure was meticulously observed and documented at both time points, namely 0 h and 24 h, using an inverted microscope.

### Transwell invasion assays

Cells in no serum basic medium were added in the upper surface of the chamber with matrigel (at 5 × 10^4^ cells per chamber). The lower chamber was filled with medium supplemented with 10% FBS. After 24 h of incubation at 37 °C, the cells on the upper surface of the membrane were removed, and the cells on the lower surface were fixed with methanol and stained with 0.1% crystal violet. The invaded cells were imaged and counted in five fields per well under a microscope.

### Gene detection

The reverse transcription quantitative PCR (RT qPCR) was performed for gene detection. Its protocols were described earlier [58]. *ACTB* served as the reference gene for normalizing gene expressions. The primers *ACTB* and *MARCKSL1* used in this study were synthesized by Takara Bio Inc. (JPN) and their sequences are provided in Table S5.

### Statistical analysis

The analysis was performed using R software (Version 4.1.2). *P*-values for the comparison between two variables were determined using a two-sided Wilcoxon rank-sum test, t test or limma test based on the data distribution. *P*-values for the correlation between two variables were determined using a Spearman or Pearson test. Error bars represent standard error. A significance level of *P* < 0.05 was considered statistically significant and denoted as “*”. “**” indicated a significance level of *P* < 0.01, while “***” indicated a significance level of *P* < 0.001, and “****” indicated a significance level of *P* < 0.0001.

## Supplementary Information

Supplementary Material 1.

## Data Availability

Raw sequencing data generated in this study have been deposited in the NCBI Sequence Read Archive (SRA) under BioProject accession PRJNA1149980 (SRA Study accession: SRP527693; SRA Run accessions: SRR30324248, SRR30324247, SRR30324236, SRR30324229, SRR30324228, SRR30324227, SRR30324226, SRR30324225, SRR30324224, SRR30324223, SRR30324246, SRR30324245, SRR30324244, SRR30324243, SRR30324242, SRR30324241, SRR30324240, SRR30324239, SRR30324238, SRR30324237, SRR30324235, SRR30324234, SRR30324233, SRR30324232, SRR30324231, SRR30324230). The data can be accessed via https://www.ncbi.nlm.nih.gov/bioproject/PRJNA1149980. The datasets supporting the conclusions of this article are included within the article and its additional files. Further inquiries can be directed to the corresponding authors.

## Abbreviations

PCa: Prostate cancer
IARC: International agency for research on cancer
GS: Gleason score
csPCa: Clinically significant PCa
isPCa: Insignificant PCa
PSA: Prostate-specific antigen
BPH: Benign prostatic hyperplasia
mpMRI: Multiparametric magnetic resonance imaging
AUCs: Area under the receiver operator characteristic curves
scRNA-seq: Single-cell RNA sequencing
TCGA: The cancer genome atlas
GEO: Gene expression omnibus
IHC: Immunohistochemical
FPKM: Fragments per kilobase million
DEGs: Differential expression genes
FBS: Foetal bovine serum
LE: Luminal epithelial cells
BE: Basal epithelial cells
NK: Natural killer
Endo: Endothelial cells
Fib: Fibroblasts
SMC: Smooth muscle cells
CNV: Copy number variation
OS: Overall survival
DSS: Disease-specific survival
ROC: Receiver operator characteristic curve
KM: Kaplan-Meier
PPI: Protein-protein interaction
ORA: Over-representation analysis
GOBPs: Gene ontology biological processes
GSEA: Gene set enrichment analysis
DRE: Digital rectal examination
MARCKS: Myristoylated alanine-rich C kinase substrates
SRA: Sequence Read Archive
FANTOM5: Functional ANnoTation Of the Mammalian genome 5
GTEx: Genotype-Tissue Expression Project
