# Supplementary material for: Integration of bulk RNA-seq data with single-cell RNA-seq data to identify urinary biomarkers derived from prostate tumor tissues
Source: BMC Urol. 2026 Aug 3;26:269. doi: 10.1186/s12894-026-02295-y (PMC13721477; doi:10.1186/s12894-026-02295-y)
Supplement: Supplementary file 1 [file 12894_2026_2295_MOESM1_ESM.docx]

**Supplementary materials**

**Supplementary tables**

**Table S1. Quality Statistics**

| **Sample ID** | **Group** | **Type** | **RIN scores** | **Clean Reads %** | **Coding Bases %** |
| --- | --- | --- | --- | --- | --- |
| Z2 | Normal | urine | 2.60 | 92.5 | 0.332531 |
| Z5 | Normal | urine | 3.00 | 81.1 | 0.261721 |
| Z6 | Normal | urine | 2.50 | 88.8 | 0.393719 |
| 8 | Normal | urine | 3.20 | 91.1 | 0.250208 |
| 11 | Normal | urine | 2.40 | 85.8 | 0.464307 |
| sc_2_N | Cancer | urine | 2.40 | 86 | 0.271131 |
| sc_3_N | Cancer | urine | 2.40 | 92.4 | 0.199898 |
| sc_4_N | Cancer | urine | 2.40 | 93.2 | 0.245341 |
| sc_6_N | Cancer | urine | 2.60 | 82.6 | 0.327706 |
| sc_7_N | Cancer | urine | 2.40 | 94.8 | 0.238605 |
| sc_2_A | Adjacent | tissues | 6.70 | 90.8 | 0.189865 |
| sc_3_A | Adjacent | tissues | 7.70 | 93.0 | 0.236527 |
| sc_5_A | Adjacent | tissues | 7.00 | 92.1 | 0.206299 |
| sc_6_A | Adjacent | tissues | 8.30 | 90.8 | 0.225216 |
| sc_7_A | Adjacent | tissues | 8.00 | 90.8 | 0.219238 |
| sc_2_T | Cancer | tissues | 8.20 | 92.8 | 0.221635 |
| sc_3_T | Cancer | tissues | 7.20 | 93.0 | 0.212035 |
| sc_5_T | Cancer | tissues | 8.80 | 95.8 | 0.230915 |
| sc_6_T | Cancer | tissues | 7.60 | 91.9 | 0.23459 |
| sc_7_T | Cancer | tissues | 7.80 | 95.8 | 0.218809 |

**Table S2. KEGG**

| **Pathway** | **No. of genes** | ***P*** | **adj.*p*** |
| --- | --- | --- | --- |
| Oxidative phosphorylation | 28 | 6.24E-26 | 1.11E-23 |
| Thermogenesis | 28 | 2.12E-19 | 1.88E-17 |
| Non-alcoholic fatty liver disease | 22 | 1.84E-16 | 1.09E-14 |
| Diabetic cardiomyopathy | 24 | 3.12E-16 | 1.39E-14 |
| Chemical carcinogenesis - reactive oxygen species | 24 | 1.57E-15 | 4.66E-14 |
| Parkinson disease | 26 | 1.35E-15 | 4.66E-14 |
| Prion disease | 25 | 2.12E-14 | 5.39E-13 |
| Huntington disease | 25 | 2.32E-13 | 5.15E-12 |
| Alzheimer disease | 26 | 7.09E-12 | 1.40E-10 |
| Amyotrophic lateral sclerosis | 25 | 1.10E-11 | 1.96E-10 |
| Metabolic pathways | 48 | 1.04E-09 | 1.68E-08 |
| Pathways of neurodegeneration - multiple diseases | 25 | 2.76E-09 | 4.10E-08 |
| Retrograde endocannabinoid signaling | 13 | 7.59E-08 | 1.04E-06 |
| Cardiac muscle contraction | 9 | 1.42E-06 | 1.81E-05 |
| Protein export | 5 | 9.10E-06 | 1.08E-04 |
| Protein processing in endoplasmic reticulum | 11 | 1.16E-05 | 1.29E-04 |
| Vibrio cholerae infection | 5 | 4.77E-04 | 4.99E-03 |
| Prostate cancer | 6 | 1.98E-03 | 1.96E-02 |

**Table S3. General information of PCa samples**

| **Patient ID** | **Sample** **ID** | **Sample type** | **Age** | **GS of biopsy** | **Detection method** |
| --- | --- | --- | --- | --- | --- |
| sc-2 | sc_2_N | PCa; Urine | 70 | 7 | RNA-seq; qPCR |
|  | sc_2_A | Tumor-adjacent; Tissue |  |  |  |
|  | sc_2_T | Tumor; Tissue |  |  |  |
| sc-3 | sc_3_N | PCa; Urine | 57 | 7 | RNA-seq; qPCR |
|  | sc_3_A | Tumor-adjacent; Tissue |  |  |  |
|  | sc_3_T | Tumor; Tissue |  |  |  |
| sc-4/sc_5 | sc_4_N | PCa; Urine | 81  76 | 7 | RNA-seq; qPCR |
|  | sc_5_A | Tumor-adjacent; Tissue |  |  |  |
|  | sc_5_T | Tumor; Tissue |  |  |  |
| sc-6 | sc_6_N | PCa; Urine | 65 | 7 | RNA-seq; qPCR |
|  | sc_6_A | Tumor-adjacent; Tissue |  |  |  |
|  | sc_6_T | Tumor; Tissue |  |  |  |
| sc-7 | sc_7_N | PCa; Urine | 70 | 7 | RNA-seq; qPCR |
|  | sc_7_A | Tumor-adjacent; Tissue |  |  |  |
|  | sc_7_T | Tumor; Tissue |  |  |  |
| sc-8 | sc-8 | PCa; Urine | 75 | 7 | qPCR |
| sc-9 | sc-9 | PCa; Urine | 68 | 6 | qPCR |
| sc-10 | sc-10 | PCa; Urine | 70 | 6 | qPCR |
| sc-11 | sc-11 | PCa; Urine | 61 | 8 | qPCR |
| sc-12 | sc-12 | PCa; Urine | 60 | 8 | qPCR |
| sc-13 | sc-13 | PCa; Urine | 65 | 7 | qPCR |
| sc-14 | sc-14 | PCa; Urine | 70 | 7 | qPCR |
| sc-15 | sc-15 | PCa; Urine | 80 | 8 | qPCR |
| sc-16 | sc-16 | PCa; Urine | 81 | 6 | qPCR |
| sc-17 | sc-17 | PCa; Urine | 72 | 7 | qPCR |
| sc-18 | sc-18 | PCa; Urine | 73 | 6 | qPCR |
| sc-19 | sc-19 | PCa; Urine | 65 | 6 | qPCR |
| sc-20 | sc-20 | PCa; Urine | 70 | 7 | qPCR |
| sc-21 | sc-21 | PCa; Urine | 66 | 7 | qPCR |
| sc-22 | sc-22 | PCa; Urine | 74 | 7 | qPCR |
| sc-23 | sc-23 | PCa; Urine | 75 | 9 | qPCR |
| sc-24 | sc-24 | PCa; Urine | 60 | 8 | qPCR |
| sc-25 | sc-25 | PCa; Urine | 62 | 6 | qPCR |
| sc-26 | sc-26 | PCa; Urine | 83 | 7 | qPCR |
| sc-27 | sc-27 | PCa; Urine | 77 | 8 | qPCR |
| sc-28 | sc-28 | PCa; Urine | 71 | 9 | qPCR |
| sc-29 | sc-29 | PCa; Urine | 68 | 6 | qPCR |
| sc-30 | sc-30 | PCa; Urine | 76 | 6 | qPCR |

GS：Gleason score

**Table S4. General information of the published scRNA-seq datasets**

| **Type** | **Description** | **Number of scRNA-seq matrices** | **ID of scRNA-seq matrices** | **Number of samples** | **Data filtering criteria** |
| --- | --- | --- | --- | --- | --- |
| Normal | Normal prostate tissues of peripheral zone (PZ) taken from the young healthy donors | 3 | GSM5252457, GSM5252459, GSM5252461 | 3 | nFeature_RNA>200&nFeature_RNA<4000&nCount_RNA<20000 |
| PCa | Carcinoma tissue taken from the prostate cancer (PCa) patients | 5 | GSM4203181(10 samples), GSM4773521, GSM4773522, GSM5353236, GSM5353237 | 13 | nFeature_RNA>200&nFeature_RNA<4000&nCount_RNA<20000 |

**Table S5. Synthesized sequences**

| Name | Sequences (5’ to 3’) |
| --- | --- |
| *ACTB* | Forward: CATGTACGTTGCTATCCAGGC |
|  | Reverse: CTCCTTAATGTCACGCACGAT |
| *MARCKSL1* | Forward: TATCCCCCAAGGGTGAAGGG |
|  | Reverse: TTCTCTTGAAGGACAGGCCG |
| siRNA-1 | S: CCCUGUGAACGGAACAGAUTT  AS: AUCUGUUCCGUUCACAGGGTT |
| siRNA-2 | S: CUUUCAAAUUGAGCGGCCUTT  AS: AGGCCGCUCAAUUUGAAAGTT |
| siRNA-3 | S: AGAGGCUAGUGCAGCCUCATT  AS: UGAGGCUGCACUAGCCUCUTT |
| siRNA-4 | S: CAGCGCUGAGCAGAAUGAGTT  AS: CUCAUUCUGCUCAGCGCUGTT |

**Figure S1. Quality control of RNA extraction and sequencing.**

**A**. RIN scores of RNAs extracted from urine exfoliated cells. **B**. Mapping distribution of urine and tissue RNA sequencing.

**Figure S2. Identifying the gene features of prostate tumor cells using scRNA-seq data.**

A. UMIs (nCount_RNA), genes (nFeature_RNA), and percent.mt per spot. **B.** Correlation between genes (nFeature_RNA) and UMIs (nCount_RNA), as well as correlation between percent.mt and UMIs (nCount_RNA). **C.** Violin plots coloured by Seurat clusters illustrating gene expression levels; UMAPs coloured by gene expression levels. **D.** inferCNV.

**Figure S3. Enrichment analysis of KEGG pathways.**

**Figure S4. *MARCKSL1* and *LYPLA1* gene expression levels in both urine and tissue samples (RNA-seq data).**

**Figure S5. *MARCKSL1* gene expression levels in seven prostate cancer cell lines (RNA-seq data).**

**Figure S6. MARCKSL1 protein and mRNA levels in prostate tissues**

**A** MARCKSL1 protein IHC staining data of prostate tissues retrieved from the Human Protein Atlas database (https://www.proteinatlas.org). **B** *MARCKSL1* mRNA expression in each cell type of prostate tissues (scRNA-seq data obtained retrieved from the Human Protein Atlas database (https://www.proteinatlas.org)).

**Figure S7. MARCKSL1 protein IHC staining data obtained from the Human Protein Atlas database (https://www.proteinatlas.org).**

**Figure S8. *MARCKSL1 transcript abundance* (RNA-seq data obtained from the GTEx and the FANTOM5 project).**

**Figure S9.** **MARCKSL1 affecting prostate tumor cellular migration and invasion.**

**A** Representative images depicting the transient transfection of PC3 and LNCap cells with FAM. **B** Bar plots depicting the quantification of *MARCKSL1* mRNA levels using qPCR analysis. **C** Images depicting wound-healing assays conducted on PC3 and LNCap cells, illustrating cellular motility subsequent to the knockdown of *MARCKSL1*. Scale bar, 50 μm. **D** Images of invasion assays using PC3 and LNCap cells, showing cell invasion after knockdown of *MARCKSL1*. Magnification, 40X and 100X.

**Figure S10. Evaluating the diagnostic potential of serum total PSA and urinary exfoliated cell *MARCKSL1* for csPCa.**

**A** Bar plots depicting the quantification of serum total PSA. **B** ROC plots demonstrating the diagnostic accuracy of serum total PSA for csPCa. **C** Spearman correlation between the quantification of urine *MARCKSL1* and Gleason scores. **D** Binary images showing the diagnostic accuracy of the diagnostic accuracy of urine *MARCKSL1* for csPCa.
